# Solid-State Fermentation of Green Coconut Husk by *Penicillium roqueforti* for the Production of a Halotolerant Xylanase

**DOI:** 10.1021/acsomega.5c10884

**Published:** 2026-05-08

**Authors:** Marise S. Carvalho, Adriana B. Pimentel, Alisson S. S. Quinto, Gabriel L. Jesus, Eliezer L. E. Santo, Floriatan S. Costa, Iana T. Emmerich, Jaqueline J. Silva, Grazielly J. Silva, Izis Rafaela A. S. Vieira, Iasnaia M. C. Tavares, Marcelo Franco

**Affiliations:** † Institute of Health Sciences, Federal University of Bahia, Salvador, Bahia 40110-100, Brazil; ‡ Biotransformation and Organic Biocatalysis Research Group, Department of Exact Sciences, 74361 Santa Cruz State University, Ilhéus 45654-370, Brazil; § Departament of Chemistry, Federal University of Paraná, Curitiba 80060-000, Brazil; ∥ Department of Biological Sciences, Santa Cruz State University, Ilhéus 45654-370, Brazil; ⊥ Department of Exact Sciences and Natural, State University of Southwest Bahia, Itapetinga 45700-000, Brazil; # Department of Human Sciences and Technologies − DCHT, State University of Bahia, Campus XXI, Ipiaú 45570-000, Brazil

## Abstract

This investigation, conducted within the framework of the circular economy, employed green coconut waste as a lignocellulosic substrate for the production of xylanase via solid-state fermentation (SSF). Enzymatic yield was optimized through a Box–Behnken experimental design, incorporating initial moisture content, incubation temperature, and fermentation duration as independent variables. Subsequent characterization of the enzyme, performed using a Doehlert design, identified optimal reaction conditions at pH 6.5, 20 °C, and 70 min, under which the xylanase exhibited notable catalytic efficiency and thermal stability. Remarkably, the enzyme demonstrated halotolerance, retaining activity in sodium chloride concentrations of up to 6 mol L^–1^, thereby indicating its potential for application under high-salinity industrial conditions. When applied to sugarcane bagasse, the xylanase facilitated a substantial release of reducing sugars, thereby confirming its efficacy in the bioconversion of agro-industrial residues. Collectively, these findings validate the use of statistical optimization strategies and underscore SSF as a sustainable and effective approach for the production of high-performance biocatalysts.

## Graphical abstract

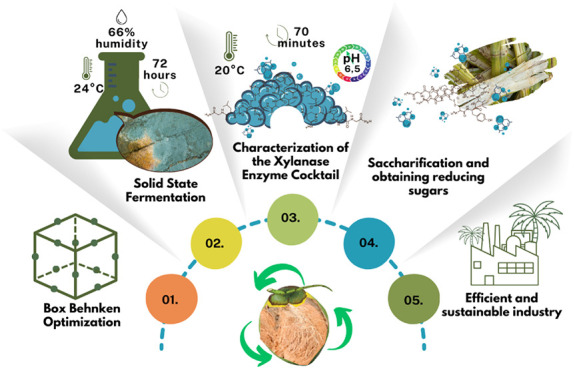

## 1 Introduction

The large-scale generation of agro-industrial residues represents a pressing environmental challenge, necessitating solutions that reconcile economic development with environmental responsibility. Such integration is facilitated by circular models of production and consumption, which prioritize waste valorization, resource-use efficiency, and the minimization of losses.^1,2^ Within this framework, lignocellulosic residues derived from the processing of green coconut (Cocos nucifera L.) warrant particular attention due to their considerable volume and slow biodegradation rate—characteristics that exacerbate the environmental impact of improper disposal practices.^3^

In 2023, global coconut production reached 64 million tonnes, with Brazil ranking fourth among the world’s leading producers.^4^ The Northeast region accounts for approximately 78% of national output, corresponding to an estimated 1.44 million tonnes annually.^5^ Given this substantial volume, the valorization of green coconut residues emerges as a promising strategy, transforming an environmental liability into a productive resource. This approach aligns with the United Nations Sustainable Development Goals (SDGs), particularly those concerning responsible production and consumption, and climate action.^6,7^

Among the technologies applicable to the valorization of agro-industrial residues, solid-state fermentation (SSF) stands out as a particularly promising approach. In this process, the residues serve as substrates, supplying essential nutrients for microbial growth, while fermentation occurs under conditions of minimal free water availability. SSF offers notable economic and environmental advantages, including the low cost of substrates, the replication of natural microbial growth conditions, and high efficiency in the conversion of waste materials into value-added biomolecules. Filamentous fungi such as *Aspergillus*, *Penicillium*, *Rhizopus*, *Fusarium*, and *Trichoderma* are among the principal microbial agents employed in SSF, owing to their robust capacity for enzyme production under low-moisture conditions.^8–16^

Enzymes obtained via SSF exhibit broad industrial applicability, functioning as biocatalysts in reactions characterized by high specificity and operating under moderate pH and temperature conditions, thereby contributing to reduced energy consumption.^17,18^ Among these biomolecules, xylanase is particularly noteworthy. Belonging to the glycoside hydrolase family, xylanase is responsible for the hydrolysis of xylan, the principal component of hemicellulose in plant cell walls. Its main variants, endo-1,4-β-xylanase (EC 3.2.1.8) and β-D-xylosidase (EC 3.2.1.37), have been extensively studied due to their relevance in the production of xylo-oligosaccharides and xylose.^19^ Furthermore, xylanase has well-established applications across various industrial sectors, including pulp and paper bleaching,^20^ juice clarification,^21^ and bioethanol production.^22^

Although solid-state fermentation (SSF) is recognized as an efficient technique for enzyme production, the utilization of green coconut husk as a lignocellulosic substrate remains underexplored, particularly with regard to xylanase synthesis. This gap highlights the need for further investigation into the biotechnological potential of this abundant yet underutilized residue. Despite the high costs typically associated with enzyme production, advances in optimization methodologies have enabled improved efficiency and cost reduction. Traditionally, the analysis of fermentative variables was conducted using univariate approaches, which are time-consuming, resource-intensive, and fail to account for interactions among factors. In contrast, multivariate experimental designs, such as Box–Behnken and Doehlert, offer greater robustness and precision, allowing for the identification of optimal conditions with a reduced number of experimental runs.^23–26^

To date, no studies have reported the integration of multivariate statistical optimization with the practical application of enzymatic extracts derived from green coconut husk, thereby underscoring the originality of the present work. Recent studies have consistently demonstrated the effectiveness of statistical designs in enhancing enzyme production via SSF. Ideal conditions of 59% moisture and 16 °C were identified for xylanase production;^27^ 60% moisture and 28 °C were reported for endoglucanase;^28^ and 90% moisture and 29 °C were obtained for β-glucosidase.^29^ These results demonstrate that statistical methodologies enable the precise identification of the most influential variables and the maximization of enzymatic yield, thereby enhancing process efficiency and economic feasibility.

In light of this context, the present study proposes an innovative approach to the valorization of green coconut husk within the framework of the circular economy. The objectives were: (i) to optimize xylanase production by *Penicillium roqueforti* ATCC 10110 using green coconut husk as substrate via SSF, employing a Box–Behnken design; (ii) to characterize the enzyme in terms of temperature, pH, and reaction time using a Doehlert design, as well as its behavior in the presence of metal ions, cosolvents, and salt tolerance; and (iii) to apply the crude enzymatic extract in the saccharification of sugarcane bagasse, aiming to assess its biotechnological potential.

## 2 Materials and Methods

### 2.1 Materials

Green coconut husks (GCH) and sugarcane bagasse (SB) were sourced from small-scale vendors of coconut water and sugarcane juice in the municipality of Ilhéus, Bahia, Brazil. The microorganism *Penicillium roqueforti* ATCC 10110 was kindly provided by the Oswaldo Cruz Foundation (FIOCRUZ, Rio de Janeiro, RJ), having been previously deposited at the National Institute for Quality Control in Health (INCQS, Rio de Janeiro, RJ) under registration number 40075, batch 079840075. All reagents employed in this study were purchased from Sigma.

### 2.2 Preparation of Residues

The residues (GCH and SB) were thoroughly washed and manually reduced in size. Subsequently, they were oven-dried at 65 °C until complete dehydration was achieved. The dried material was then ground and stored in airtight containers for subsequent experimental use.

### 2.3 Inoculum Preparation

The inoculum was prepared using Potato Dextrose Agar (PDA) medium and incubated at 28 °C in a bacteriological incubator (SL700, Solab, Brazil) for a period of 7 days. Following incubation, the sporulated culture was scraped using sterile glass beads and suspended in a Tween 80 solution (0.01% v/v). All materials used in the process were previously sterilized in a vertical autoclave at 121 °C for 15 min. The spore suspension was collected in Erlenmeyer flasks, and for spore concentration determination, a 0.1 mL aliquot was transferred to an Eppendorf microtube and counted under a binocular microscope (Model L1000, Bioval, Montpellier, France) using a Neubauer counting chamber.

### 2.4 Solid-State Fermentation

Solid-state fermentations were carried out using 5 g of green coconut husk (GCH), placed in 125 mL Erlenmeyer flasks previously sterilized in a vertical autoclave at 121 °C for 15 min. Following autoclaving and subsequent cooling, inoculation was performed at a concentration of 10^7^ spores. Moisture content was adjusted by the addition of distilled water, while the intrinsic water content of the substrate was determined using an infrared moisture analyzer (ID200, MARTE, São Paulo, Brazil). The Erlenmeyer flasks were incubated in a temperature-controlled BOD chamber (TE-371, Tecnal, Piracicaba, Brazil), with fermentation time defined according to the parameters established by the statistical design.

### 2.5 Optimization of Xylanase Production

The optimization of xylanase production was conducted using a Box–Behnken experimental design. Within the established experimental matrix, three independent variables were assessed: initial moisture content (U, %), incubation temperature (T, °C), and fermentation time (t, h), each evaluated at three distinct levels. The design comprised 12 unique combinations of variables, along with three replicates at the central point, which were included to estimate experimental error, resulting in a total of 15 trials, as detailed in Table 1.

**Table 1:** Box–Behnken Matrix for the Independent Variables: Time (T, H), Temperature (T, °C), and Moisture Content (U, %), and the Dependent Variable Xylanase Activity (XIL, U/g) Obtained from the Enzymatic Extract Produced by *P. roqueforti* ATCC 10110 Cultivated on 5 g of Green Coconut Husk^a^

Experiment	Time (h)	Temperature (°C)	Humidity (%)	Xylanase enzymatic activity (U/g)
1	24 (−1)	18 (−1)	65 (0)	11.60 ±0.09
2	120 (1)	18 (−1)	65 (0)	10.79 ± 0.02
3	24 (−1)	32 (1)	65 (0)	10.25 ± 0.07
4	120 (1)	32 (1)	65 (0)	9.46 ± 0.05
5	24 (−1)	25 (0)	50 (−1)	10.06 ± 0.02
6	120 (1)	25 (0)	50 (−1)	11.32 ± 0.09
7	24 (−1)	25 (0)	80 (1)	9.71 ± 0.05
8	120 (1)	25 (0)	80 (1)	11.14 ± 0.09
9	72 (0)	18 (−1)	50 (−1)	10.25 ± 0.03
10	72 (0)	32 (1)	50 (−1)	9.19 ± 0.23
11	72 (0)	18 (−1)	80 (1)	9.65 ± 0.05
12	72 (0)	32 (1)	80 (1)	11.60 ± 0.09
13	72 (0)	25 (0)	65 (0)	12,55 ± 0.08
14	72 (0)	25 (0)	65 (0)	13,16 ± 0.08
15	72 (0)	25 (0)	65 (0)	12,98 ± 0.08

a The design presents both actual and coded values, with the latter shown in parentheses.

The response variable analyzed in the design was xylanase activity (XIL, U/g), quantified in the crude extract obtained following the fermentation process. Experimental data were subjected to regression analysis, and mathematical models were fitted using enzymatic activity (U/g) as the dependent variable. The adequacy of the fitted models was evaluated based on the coefficient of determination (R^2^) and the significance of lack-of-fit. Statistical processing of the data was performed using Statistica 12 software (StatSoft, Tulsa, USA), with a confidence level of 95% (p = 0.05).

### 2.6 Enzymatic Extract Preparation

Following the fermentation period, the Erlenmeyer flasks were removed from the BOD incubator and supplemented with 50 mL of sterile distilled water. The contents were then transferred to an orbital shaker (Model SL 222, Solab, Piracicaba, São Paulo, Brazil) and agitated at 200 rpm, 35 °C, for 10 min. Solid residues were separated by simple filtration, followed by mechanical pressing. The resulting liquid was centrifuged at 3000 rpm for 20 min using a benchtop centrifuge (Model CT-6000R, Cientec, Porto Alegre, Rio Grande do Sul, Brazil). The supernatant, hereafter referred to as the Enzymatic Extract (EE), was utilized for enzymatic activity assays and subsequent experimental analyses.

### 2.7 Enzymatic Assay

Enzymatic assays were performed in accordance with the standardized procedures recommended by the IUPAC Biotechnology Commission.^30^ The quantification of reducing sugars released during the reaction was based on the 3,5-dinitrosalicylic acid (DNS) method, wherein 3,5-dinitrosalicylic acid is reduced to 3-amino-5-nitrosalicylic acid, concomitant with the oxidation of aldehyde or ketone groups to carboxylic acids, resulting in the development of an intense orange-brown coloration.^31^ Reactions were conducted in test tubes containing 0.25 mL of xylan solution (1%), 0.25 mL of citrate buffer (0.5 M, pH 4.8), and 0.25 mL of EE. For control samples, xylan and EE were respectively replaced by buffer. All samples were incubated at 50 °C for 10 min. Following incubation, 0.5 mL of 3,5-dinitrosalicylic acid was added to each tube, which were then heated in a water bath at 100 °C for 5 min. The tubes were subsequently cooled, and volumes adjusted by the addition of 6.5 mL of distilled water. Absorbance readings were taken at 540 nm using a UV–Vis spectrophotometer (SF200DM-UV Vis, Bel Photonics, Osasco, Brazil), and values were compared against a standard xylose calibration curve (1–10 μmol/mL).

### 2.8 Enzymatic Characterization

#### 2.8.1 Characterization via Multivariate Analysis

To evaluate the influence of the independent variables—pH, reaction time (t, min), and temperature (T, °C)—on the activity of xylanase present in the enzymatic extract (EE), and to determine its optimal operating conditions, a Doehlert experimental design was employed. The design considered three factors, assessed at seven, five, and three levels for time, temperature, and pH, respectively.

The experimental matrix comprised 15 trials, including three replicates at the central point, which were used to estimate experimental error (Table 2). Regression models were fitted and analyzed using analysis of variance (ANOVA), allowing for the identification of factor interactions and standardized effects. The goodness-of-fit of the models was evaluated using the coefficient of determination (R^2^).

**Table 2:** Doehlert Matrix for the Enzymatic Characterization of Xylanase Obtained from the Fermentation Process.^a^

Experiment	Time (min)	Temperature (°C)	pH	Residual activity (%)
1	60	60	6	82.30 ± 0.02
2	90	50	6	25.53 ± 0.04
3	70	50	9	0.24 ± 0.032
4	60	20	6	115.95 ± 0.06
5	30	30	6	52.14 ± 0.05
6	50	30	3	3.61 ± 0.02
7	30	50	6	74.57 ± 0.04
8	50	50	3	0.24 ± 0.03
9	90	30	6	99.08 ± 0.05
10	80	40	3	50.47 ± 0.05
11	70	30	9	61.66 ± 0.05
12	40	40	9	38.44 ± 0.03
13	60	40	6	100.00 ± 0.04
14	60	40	6	115.95 ± 0.05
15	60	40	6	107.98 ± 0.05

a Independent variables: reaction time (T, min), temperature (T, °C), and pH; dependent variable: residual xylanase activity (%).

All statistical calculations and analyses were performed using Statistica 12 software (StatSoft, New York, USA).

#### 2.8.2 Influence of Metal Ions and Organic Compounds on Enzymatic Activity

The effect of various metal ion species and organic compounds on xylanase activity was assessed using 0.2 M solutions of the following reagents: copper­(II) sulfate, sodium sulfate, iron­(II) sulfate, cobalt­(II) sulfate, magnesium sulfate, lead­(II) acetate, zinc acetate, magnesium chloride, sodium carbonate, calcium carbonate, and aluminum nitrate. In addition, the following solvents were tested: EDTA (ethylenediaminetetraacetic acid), Triton, Trolox X-100 (t-octylphenoxypolyethoxyethanol), and lactose. The prepared solutions were incubated in contact with the enzymatic extract (EE), and xylanase activity was determined according to the procedure described in Section 2.6. Enzymatic activity was expressed as relative activity (%), with the activity of xylanase in the absence of additives considered as 100%.

#### 2.8.3 Influence of Cosolvents on Enzymatic Activity

The impact of cosolvents on xylanase activity was investigated using analytical-grade ethanol (99.5%), hexane, methyl ether, analytical-grade dichloromethane (1320 g, 100%), and acetone. Solutions were prepared at concentrations of 10%, 20%, and 30%, with the final volume adjusted to 4 mL using citrate buffer (0.1 M, pH 6.5). Samples were incubated in contact with the EE, followed by enzymatic activity determination as outlined in Section 2.6. Xylanase activity in the absence of cosolvents was used as the reference (100%), and results were expressed as relative activity.

#### 2.8.4 Effect of NaCl Addition on Enzymatic Activity

To evaluate the halotolerance of xylanase, the EE was incubated in NaCl solutions ranging from 0.5 to 6 M for 30 min. Aliquots from each concentration were subsequently collected for enzymatic activity determination, following the procedure described in Section 2.6. Enzymatic activity was expressed as relative activity (%), with the activity of xylanase in the absence of NaCl considered as 100%.

### 2.9 Application of the Enzymatic Extract in Sugarcane Bagasse Saccharification

The enzymatic saccharification of sugarcane bagasse (SB) was performed using an orbital shaker (Model 501-D, Ethik Tecnologia, Vargem Grande Paulista, São Paulo, Brazil) equipped with a thermostatic bath maintained at 50 °C for 6 h. The reaction medium consisted of 10 mL of enzymatic extract (EE), 10 mL of citrate buffer (0.1 M, pH 6.5), and 100 mg of SB. In addition to this system, two further experimental media were prepared containing Ca^2+^ ions at concentrations of 2 mmol and 10 mmol, respectively. A control medium was prepared without the addition of EE, serving as a reference for evaluating the effects of enzymatic saccharification. After 6 h of reaction, aliquots were collected from the experimental systems and subjected to sugar identification and quantification via high-performance liquid chromatography (HPLC).

#### 2.9.1 Sugar Determination by HPLC

The separation, identification, and quantification of sugars were carried out using high-performance liquid chromatography (HPLC) on a Shimadzu AUW 220D system (Shimadzu, DGU 20A), equipped with a refractive index detector (Shimadzu RID-20A). The analysis was conducted using a Supelcogel Ca column (30 cm × 7.8 mm, Sigma-Aldrich, Agilent, USA), maintained at 80 °C and operated at a flow rate of 0.6 mL/min. Sample extracts were filtered through polyester membranes (0.20 μm, Chromafil PET 20/25, Macherey–Nagel, Düren, Germany) and directly injected into the chromatographic column (20 μL). The mobile phase consisted of Milli-Q water. Compound identification was performed by comparing retention times and ultraviolet (UV) absorption spectra of chromatographic peaks with those of analytical standards. Sugar quantification was conducted using the external standard method, with peak height employed as the analytical parameter. Calibration curves were constructed by diluting standards in Milli-Q water, covering six concentration points (0.1 to 1 mg/L) for sucrose, glucose, and fructose.

## 3 Results and Discussion

### 3.1 Optimization of Xylanase Production

Xylanase enzymatic activity was observed under all experimental conditions evaluated (Table 1), with variations reflecting the combined influence of fermentation time, temperature, and moisture content on enzyme production. This behavior may be attributed to the ability of *Penicillium roqueforti* ATCC 10110 to grow on diverse natural substrates and secrete enzymes capable of degrading compounds present in lignocellulosic materials.^32,33^ Enzymatic activity values ranged from 9.19 U/g (72 h, 32 °C, 50% moisture) to 13.16 U/g (72 h, 25 °C, 65% moisture), indicating that intermediate temperature and moisture conditions favored enzyme production. Biologically, shorter fermentation times tend to reflect the exponential growth phase, during which energy is allocated to biomass formation, whereas prolonged incubation may reduce enzyme yield due to substrate depletion and the accumulation of inhibitory metabolites.

This pattern suggests that the combination of factors exerts a greater impact than the isolated variation of individual variables, reinforcing the importance of experimental design in elucidating such interactions. Similar findings have been reported in solid-state fermentations using agro-industrial residues, where moderate cultivation conditions proved more conducive to xylanase production.^8,10,34^

The green coconut husk (GCH) likely contributed to these results due to its hemicellulosic fraction, which may reach up to 18%. Partial hydrolysis of this fraction during SSF releases xylo-oligosaccharides and xylose, which act as inducers in filamentous fungi by activating transcriptional regulators of the Xln *R* type (or homologues). These regulators bind to the promoters of xylanase and other hemicellulase genes, enhancing enzyme secretion while simultaneously alleviating glucose-mediated catabolite repression. This mechanism is consistent with reports in *Penicillium* and related genera.^35–37^ In addition to its inductive effect, the porosity and water retention capacity of GCH may improve oxygen diffusion and the availability of bound water during SSF—conditions known to favor the secretion of extracellular enzymes^36^ Compared to other residues such as wheat bran or sugarcane bagasse, GCH offers regional availability, low cost, and sufficient hemicellulose content to support xylanase production, thereby reinforcing its technological appeal.

The fitted mathematical models were evaluated for their predictive capacity. The quadratic model exhibited the best performance (R^2^ > 0.80) and a nonsignificant lack-of-fit (*p* > 0.05), indicating satisfactory predictive ability within the experimental domain. Despite its performance, the quadratic model presents limitations, as it penalizes extreme values through the A^2^, B^2^, and C^2^ terms and assumes uniform curvature.

Figure 1A illustrates the standardized effects of the variables and their interactions. The quadratic term for temperature (B^2^) was the most significant, followed by moisture (C^2^) and time (A^2^), all with negative coefficients, indicating that deviations from the central condition compromise the response. From a bioprocess standpoint, the significant quadratic effect of temperature (B^2^) highlights its essential function in fungal metabolism and enzyme biosynthesis. Deviations from the optimal temperature range can negatively impact microbial growth, enzymatic expression, and protein stability. The quadratic effect of moisture (C^2^) relates to its influence on substrate swelling, oxygen transfer, and nutrient diffusion, all of which are essential for fungal activity in solid-state systems. Both insufficient and excessive moisture can hinder microbial development and enzyme secretion. Similarly, the quadratic effect of time (A^2^) suggests that enzyme production peaks at a specific fermentation stage, after which prolonged incubation may result in nutrient depletion, the accumulation of inhibitory metabolites, or proteolytic degradation of the enzyme.^38^ The B × C interaction was both significant and positive, suggesting local synergism between temperature and moisture, wherein moderate moisture mitigates conformational stress induced by temperature—preserving hydration water and active site flexibility—while moderate temperature reduces excessive substrate plasticization at high moisture levels.^39^

**Figure 1:**
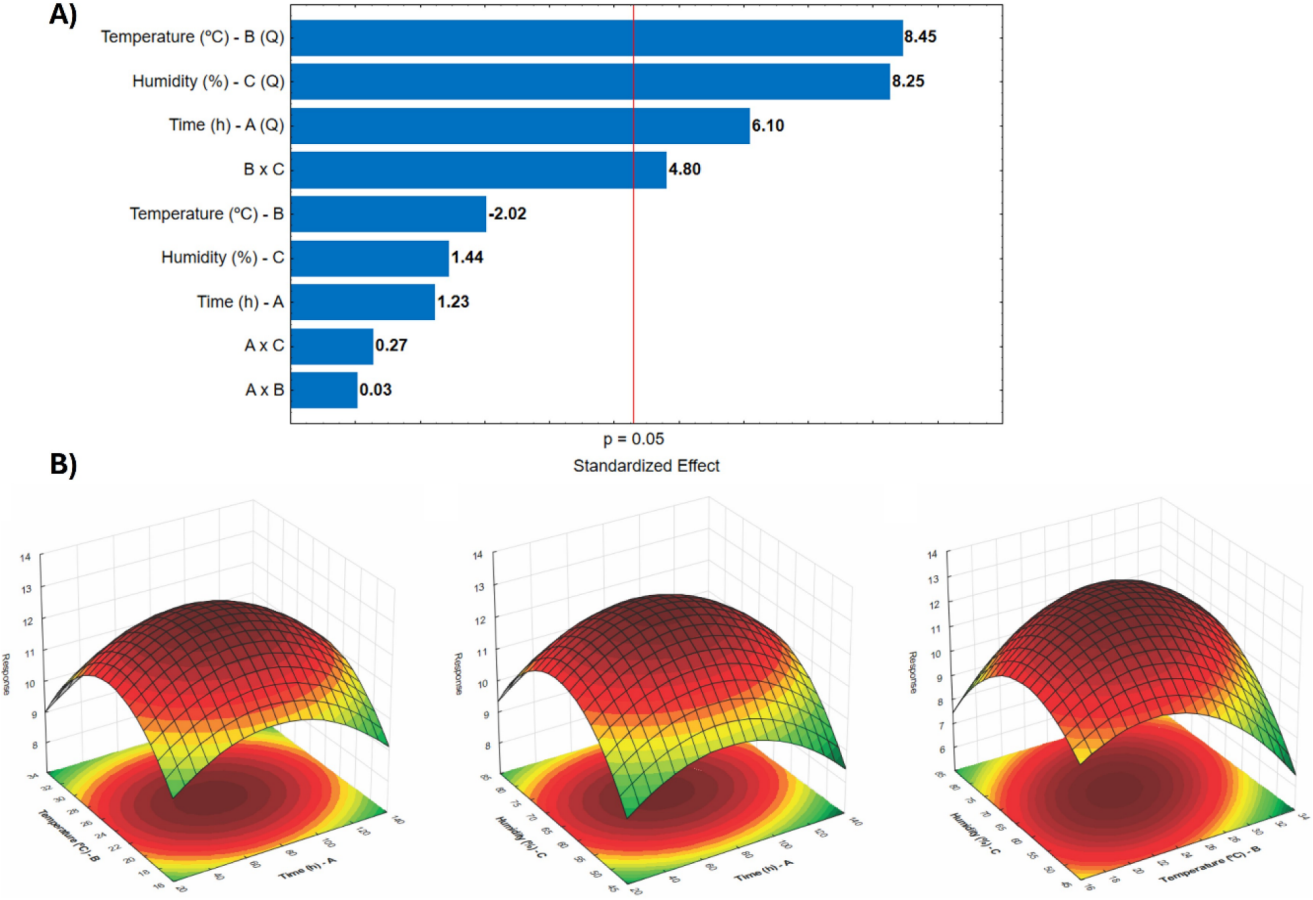
Representation of the results of the Box–Benhken experimental design for the influential variables of fermentation: A) Pareto chart indicating the significance of the terms of the variables time (A), temperature (B) and humidity (C), as well as the interaction of these terms. B) Contour plot obtained from the model.

Based on the fitted model (eq 1), the contour plots presented in Figure 1B indicated an optimal point at 24 °C, 66% moisture, and 75 h, with an estimated xylanase activity of 12.91 U/g. Experimental validation, performed in triplicate, yielded an observed activity of 13.85 ± 0.94 U/g. Although the measured value was slightly higher than the predicted one, the difference was not statistically significant (p = 0.229), which is consistent with intrinsic variations inherent to solid-state fermentation (SSF), such as local heterogeneity in moisture and porosity, microgradients in temperature, and oxygen availability.

1 $$\mathrm{Enzymatic Activity}\left(U/g\right)=12.90_{\left(\pm 0.56\right)}+0.75_{\left(\pm 0.48\right)}B\times \mathrm{C - 0}.99_{\left(\pm 0.50\right)}\mathrm{A² - 1}.38_{\left(\pm 0.50\right)}\mathrm{B² ‐ 1}.35_{\left(\pm 0.50\right)}\mathrm{C²}$$

It is noteworthy that the validated point lies near, but not exactly at, the central points of the design; thus, minor asymmetries in the response surface may account for local overestimation by the model or, alternatively, reflect a slightly superior actual performance relative to the predicted optimum.

When contextualized in the broader literature, the xylanase activity obtained under the validated optimal conditions (13.85 ± 0.94 U/g) demonstrates competitive performance in relation to other lignocellulosic substrates reported for solid-state fermentation (SSF) systems. Comparative evaluations indicate that residues such as açaí, sawdust, and wheat or corn bran typically exhibit lower activities, often remaining close to 1.9 U/g even after optimization,^40^ while pomegranate peel reaches approximately 3.91 U/g,^41^ values considerably lower than those observed in the present study. Studies using agricultural byproducts, such as sugarcane bagasse combined with coconut husk^9^ or using only coffee husk,^27^ report moderate activities of 12.15 and 13.20 U/g, respectively, closely aligning with the response validated here. Although higher activities have been documented for substrates such as sugarcane bagasse and agave (26.6 U/g),^42^ the performance obtained with green coconut husk stands out for matching or surpassing many recent reports, using an abundant and low-cost agro-industrial waste. Together, these comparisons reinforce that the optimized conditions identified in this study allow for the efficient exploitation of coconut husk as a robust substrate for the sustainable production of xylanase.^44^

In summary, the experimental design proved effective in mapping the response surface and identifying robust operational conditions. From the perspective of substrate feasibility, green coconut husk (GCH) demonstrates competitive potential compared to other lignocellulosic residues due to its hemicellulose content, which can release inducers such as xylo-oligosaccharides and xylose; its favorable physical matrix for solid-state fermentation (SSF), offering water retention and porosity; its broad availability and low cost; and its experimental performance, with maximum enzymatic activity within the studied domain exceeding the overall mean.

Future studies may explore expanded ranges of temperature (B) and moisture (C) to assess surface asymmetry and incorporate cubic terms, as well as directly compare GCH with other residues under identical operational conditions. Such investigations should quantify productivity, yield per unit mass of substrate, and pretreatment costs—critical parameters for industrial decision-making.

### 3.2 Enzymatic Characterization

#### 3.2.1 Characterization via Multivariate Analysis

The enzymatic characterization of xylanase was performed using a Doehlert experimental design, enabling multivariate optimization of temperature, pH, and reaction time. Data on residual enzymatic activity are presented in Table 2. It was observed that the enzyme maintained high residual activity at elevated temperatures, provided the ideal pH range was preserved. This behavior is related to the preservation of the native structure, supported by specific noncovalent interactions, such as ion pairs and salt bridges, which stabilize the folded state of the protein. Additionally, the presence of particular structural elements and the conservation of the active site contribute to maintaining the enzyme’s functional conformation under conditions of thermal stress.^43^

The quadratic model fitted to the data exhibited high accuracy (R^2^ > 0.97), allowing for the determination of optimal conditions and the relative influence of each variable (Figure 2A). The multiparametric equation obtained for the residual enzymatic activity (Y), as a function of reaction time (A), temperature (B), and pH (C), in coded values, can be expressed as

2 $$\mathrm{Enzymatic Activity}\left(U/g\right)=107.98_{\left(\pm 7.03\right)}-22.89_{\left(\pm 6.09\right)}B-47.99_{\left(\pm 12.18\right)}A\times B-40.11_{\left(\pm 11.12\right)}A\times C-42.93_{\left(\pm 8.34\right)}\mathrm{A²}-71.18_{\left(\pm 7.03\right)}\mathrm{C²}$$

**Figure 2:**
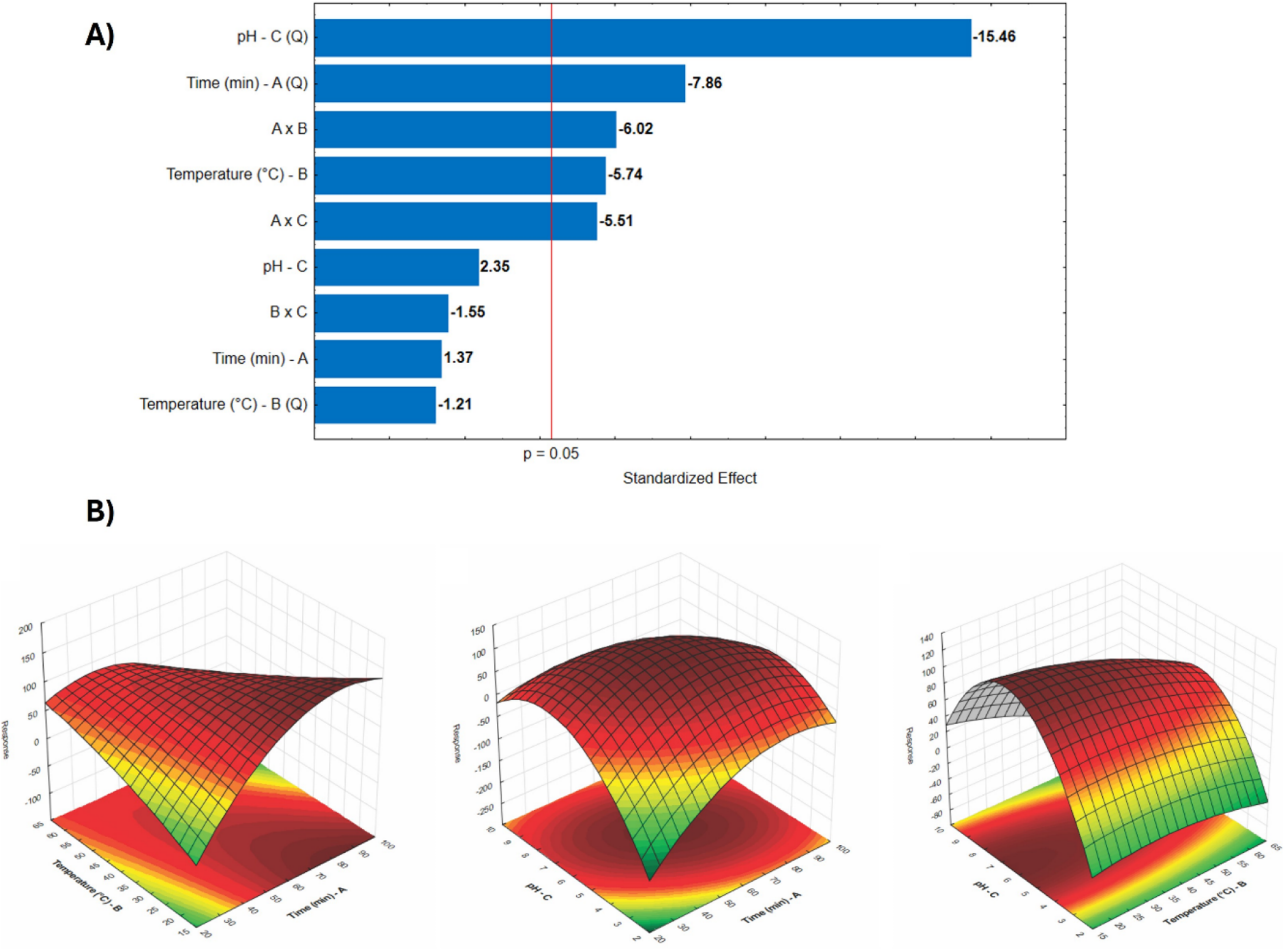
Representation of the results of the Doehlert experimental design for the characterization of xylanase. A) Pareto chart indicating the significance of the terms of the variables time (A), temperature (B), and pH (C), as well as the interaction of these terms. B) Contour plot obtained from the model.

Among the factors, pH (variable C) had the greatest impact, followed by reaction time (A). Additionally, the interactions between time and temperature (A × B), and between time and pH (A × C), were statistically significant and exhibited negative effects. Taken together with the negative quadratic terms for A, B, and C, these results indicate that simultaneous deviations from the optimal conditions of time, temperature, and pH lead to a significant decrease in enzymatic activity, particularly when prolonged reaction times are paired with higher temperatures or pH values that fall outside the ideal range. From a biochemical perspective, the negative quadratic coefficient related to reaction time (A^2^) suggests that, after a certain duration, the enzyme’s persistence in the reaction mixture promotes gradual denaturation, autoprolysis, or aggregation, which in turn reduces the available active fraction. Additionally, the negative interactions A × B and A × C imply that these processes are further accelerated when extended reaction times are combined with elevated temperatures or pH values far from the optimum. This is consistent with the cooperative nature typically associated with thermal denaturation and the destabilization of electrostatic interactions at the protein surface.^38^

This indicates that reducing these variables tends to enhance enzymatic activity, which is consistent with the understanding that extreme pH values or prolonged reaction times may lead to enzyme denaturation, while elevated temperatures accelerate the loss of functional structure.

From a biochemical standpoint, pH directly influences the ionization state of critical amino acid residues such as aspartic acid, glutamic acid, histidine, lysine, and arginine, which are responsible for stabilizing interactions including salt bridges, hydrogen bonds, and essential electrostatic forces. Deviations in pH may lead to protonation or deprotonation of these residues, thereby disrupting such interactions. Moreover, optimal pH ensures proper alignment between the active site and substrate, facilitating catalysis, whereas pH values outside this range may result in partial unfolding or irreversible inactivation of the enzyme.^45,46^

In the specific case of this xylanase, the optimal conditions were identified as pH 6.5 and a temperature of 20 °C, with a reaction time of 70 min, yielding a predicted residual activity of 134.05%, which was experimentally validated without significant deviation. These values indicate a stable and efficient enzyme under mild conditions, suggesting its potential applicability in industrial processes with lower energy demands, such as biofuel production, food processing, and the paper industry.^47,48^

The results obtained underscore the effectiveness of multivariate methods in studying the biochemical characteristics of enzymes, in contrast to the univariate approach. While univariate analysis still widely employed, focuses on the isolated effect of each variable, multivariate designs allow simultaneous investigation of factor interactions, such as temperature, pH, and time, providing more comprehensive insights and reducing the number of required experiments. Study^26^ compared both approaches, demonstrating that multivariate analysis not only identifies synergistic effects among variables but also enhances experimental design efficiency and result resolution. Furthermore, the experimental design adopted in this study enabled the evaluation of factors at differentiated levels a feature highlighted by as particularly valuable, as it allows prioritization of specific variables such as enzyme reaction time, which warrants special attention.^49^

#### 3.2.2 Analysis of the Influence of Metal Ions and Organic Compounds on Enzymatic Activity

In this study, xylanase activity was positively influenced by the majority of the evaluated metal ion species (Figure 3), an effect that may be attributed to the ability of certain ions to stimulate the enzymatic active site. The greatest increase in activity was observed in the presence of Ca^2+^, reaching 150% relative activity. This behavior can be explained by the fundamental role of calcium in structural stabilization and catalytic efficiency of xylanase, as it protects critical interfacial regions involved in substrate recognition and preserves the integrity of xylan-binding subsites.^50^

**Figure 3:**
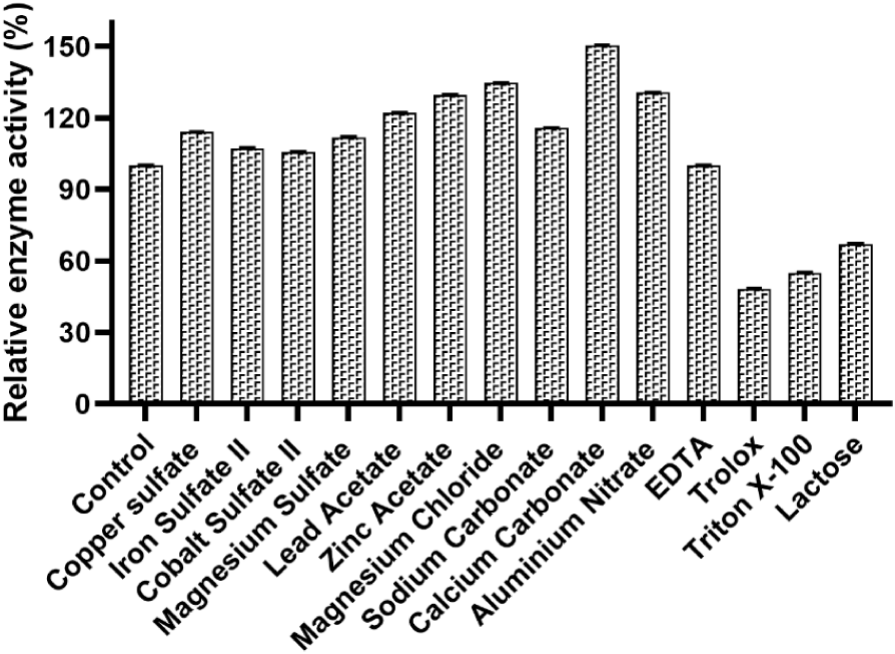
Influence of metals and solvents on the enzymatic activity of xylanase from *P. roqueforti* ATCC10110 in green coconut shell. The activity was expressed as relative activity (%) considering as 100%; the activity obtained without any metal salt or solvent.

Similar results have been reported for xylanases from *Aspergillus niger* GIO and *Bacillus* sp. (BA), produced from alkali-pretreated maize straw residue,^51^ as well as for xylanase from *Penicillium roqueforti* ATCC 10110 obtained from yellow cajá fruit residue.^10^ This dependence on Ca^2+^ is consistent with the necessity of maintaining the geometry of the active site, as its absence may impair substrate recognition.^52^

In addition to calcium, other ions such as Mg^2+^, Al^3+^, Zn^2+^, and Pb^2+^ also promoted a significant increase in xylanase activity, in contrast to findings reported for xylanase from *Aspergillus oryzae* ATCC 10124 produced from mixed residues.^9^ Previous studies indicate that Mg^2+^ and Zn^2+^ often act as cofactors, stabilizing the three-dimensional structure through interactions with charged residues, particularly Asp and Glu, thereby preserving the proper conformation of the active site and facilitating substrate orientation for catalysis.^53^ Similarly, Fe^2+^ also exhibited a positive effect in certain systems, reinforcing the role of divalent metal ions in maintaining conformational stability and enzymatic functionality. These variations in response to different ions may be attributed to the structural specificity of xylanases, and the identification of distinct activators may broaden their potential for application in diverse industrial processes.^11^

Conversely, most of the organic compounds tested exerted an inhibitory effect on xylanase activity. Trolox exhibited the highest degree of inhibition, reducing activity by more than 50%, a result contrary to that observed for xylanase from *P. roqueforti* ATCC 10110, which showed an activating effect in the presence of the same compound.^10^ The inhibition caused by Trolox and Triton X may be attributed to the disruption of hydrophobic interactions essential for enzyme structural stability. Xylanases generally exhibit low surface hydrophobicity and rely heavily on hydrophobic interactions to maintain their active conformation and ensure efficient substrate recognition. Trolox, a hydrophilic analogue of vitamin E, may sequester these interactions or alter the overall hydrophobic balance, resulting in a less stable conformation and, consequently, reduced enzymatic activity. Similarly, nonionic surfactants such as Triton X-100 may encapsulate hydrophobic protein groups within micelles, disrupting internal interactions that support the native structure. Although such surfactants may, in some systems, reduce nonspecific enzyme adsorption to hydrophobic surfaces and preserve activity, the absence of these interactions—as observed in xylanases with low surface hydrophobicity—may lead to the opposite effect, diminishing catalytic efficiency.^53,54^

Among the compounds evaluated, only EDTA did not exhibit an inhibitory effect, maintaining 100% relative activity. Nevertheless, several studies have identified EDTA as a potential inhibitor of xylanases, possibly due to its ability to chelate ions essential for structural stability.^51,52^

The results obtained in this study demonstrate that xylanase exhibits high tolerance—and even stimulation—in the presence of certain metal ions such as Ca^2+^, Mg^2+^, and Zn^2+^, corroborating literature that describes the structural and stabilizing roles of these cations in the three-dimensional conformation of enzymes.^50,55^ This profile suggests promising potential for industrial applications in processes operating in mineral-rich environments, such as pulp pretreatment and bleaching,^56^ enzyme-enriched feed production,^57^ and lignocellulosic waste bioconversion.^9,27^ On the other hand, the observed sensitivity to organic compounds such as Trolox and Triton X-100 indicates that enzyme performance may be compromised in the presence of agents that interfere with intramolecular hydrophobic interactions, highlighting the importance of optimizing reaction medium composition to preserve catalytic activity.^53,54^

#### 3.2.3 Analysis of the Effect of Cosolvents on Enzymatic Activity

The activity of fungal xylanase was evaluated in the presence of varying concentrations of organic solvents, revealing that these agents may act either as enzymatic activators or inhibitors depending on their physicochemical properties and the concentrations employed (Table 3). Organic solvents influence enzymatic activity through several molecular mechanisms: they may alter the polarity and rigidity of the enzyme’s microenvironment, displace the hydration shell essential for protein stability, and modulate the three-dimensional conformation via interactions with specific amino acid residues. These changes may either enhance the alignment of the active site with the substrate or compromise catalytic efficiency.^58,59^

**Table 3:** Influence of Cosolvents on the Xylanase Activity of *P. roqueforti* ATCC 10110 in Green Coconut Shell^a^

	Relative activity (%)			
Concentration (%)	Alcohol	Hexane	Dichloromethane	Acetone
10	52.40 ± 0.06	88.67 ± 0.02	134.36 ± 0.05	3.36 ± 0.09
20	58.45 ± 0.05	71.20 ± 0.03	94.72 ± 0.12	2.69 ± 0.09
30	113.53±0.03	58.45 ± 0.03	65.83 ± 0.04	0.05 ± 0.01

a The activities were expressed in relative activity considering as 100%; the activity obtained without the addition of cosolvents.

Among the solvents tested, ethanol stood out positively, increasing the relative activity of xylanase at all concentrations, with a maximum value of 113.53% at 30% (v/v). Studies such as those on G2 xylanase have reported similar stability in the presence of ethanol and other alcohols (methanol, isopropanol), although the extent of tolerance varies depending on the xylanase source and experimental conditions.^60^ Comparable results were reported by ref.^61^, demonstrating the maintenance of xylanase activity in the presence of ethanol. This ethanol resistance may be attributed to the enzyme’s ability to preserve its hydrophobic core, tolerating moderate disruptions in the hydration network, an advantageous trait for applications in simultaneous saccharification and fermentation (SSF) processes in second-generation biofuel production.^58^

Less polar solvents such as hexane, and those of intermediate polarity like dichloromethane, also promoted increased activity at low concentrations, possibly due to enhanced solubility of hydrophobic substrates and suppression of secondary aqueous reactions. However, at higher concentrations, these solvents tend to destabilize hydrophobic interactions essential for enzymatic conformation. Acetone, a polar aprotic solvent, did not enhance activity and instead reduced it across all concentrations tested, corroborating studies that highlight its potential to strip the enzyme’s bound water layer and disrupt hydrogen bonds critical to structural stability.^62,63^

From a molecular perspective, polar residues such as serine, threonine, glutamine, and aspartic acid are more sensitive to changes in hydration, while hydrophobic residues (leucine, isoleucine, phenylalanine) contribute to maintaining the enzyme’s internal structure. Solvent interactions may induce rearrangements in the disulfide bond network (cysteine), compromising enzyme rigidity and activity. Less polar solvents like hexane exert a lower impact on these arrangements than more polar solvents such as acetone.^64^

Furthermore, the hydration shell plays a fundamental role in the conformational stability and mobility of xylanase. Water bound to the protein supports electrostatic interactions and hydrogen bonding, enabling controlled flexibility at the active site. When polar solvents remove this layer, mobility may become either restricted or excessive, ultimately leading to reduced catalytic efficiency. Solvents that preserve part of this layer or interact selectively with it may, paradoxically, maintain or even enhance enzymatic activity.^64^

In summary, the results indicate that this xylanase exhibits notable tolerance to ethanol and, to a lesser extent, hexane and dichloromethane, while acetone consistently acts as an inhibitor. This profile suggests suitability for industrial environments of low to moderate polarity, such as biomass extraction or pretreatment processes, and helps identify solvents most compatible with enzymatic activity preservation. Solvents like ethanol are clearly more appropriate for biofuel systems and fermentation processes, whereas polar aprotic solvents should be avoided.

#### 3.2.4 Effect of NaCl Addition on Enzymatic Activity

Halotolerance is a characteristic of certain enzymes that enables them to retain catalytic activity even in environments with high salt concentrations, such as NaCl. This property is of considerable interest for industrial and biotechnological applications operating under hypersaline conditions, including seafood processing, aquaculture feed production, industrial effluent treatment, and bioremediation of saline–alkaline soils.^65^

In the present study, the evaluated xylanase retained 69.8 ± 2.1% of its residual activity when exposed to the highest tested NaCl concentration (6 M) and 94.7 ± 1.5% activity at 4 M NaCl (Figure 4). These values suggest high performance at elevated salt concentrations; however, the activity profile relative to NaCl concentration showed significant and nonsystematic fluctuations across the assessed range. No clear pattern of increase or decrease was observed with rising ionic strength. The highest value recorded at 4 M NaCl should be interpreted with caution, as it falls within the limits of experimental variability and does not independently establish a well-defined optimum in comparison to lower concentrations. These results indicate high enzymatic stability under extreme salinity conditions.

**Figure 4:**
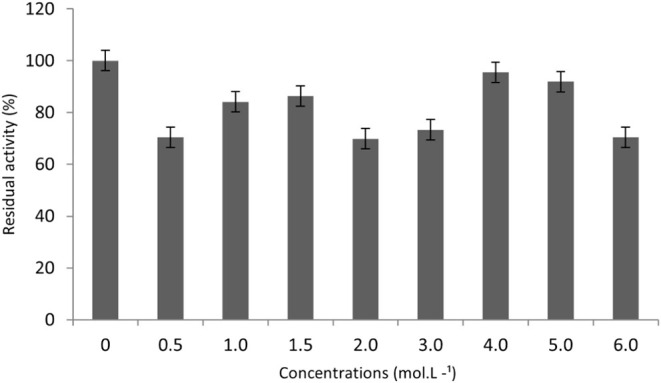
Effect of different NaCl concentrations on xylanase activity. The experiments were performed in triplicate, and the results are presented in relative activity considering the reaction without the addition of NaCl or 100%.

Comparatively, marine-derived halotolerant xylanases described by Qeshmi^66^ maintained approximately 80% activity at 4 M NaCl, while others from halophilic microorganisms achieved 90–100% activity at concentrations above 3 M,^65^ suggesting that the enzyme investigated in this study exhibits competitive performance. The data do not show a specific optimum activity at any particular NaCl concentration. Instead, they indicate that the enzyme can maintain significant residual activity across the range of concentrations tested, including at 6 M NaCl, which aligns with halotolerant behavior.

The stability of enzymes under high salinity conditions can be attributed to the characteristic structural composition of halotolerant proteins. The presence of Na^+^ and Cl^–^ ions in the reaction medium may promote electrostatic interactions among charged amino acid residues located on the protein surface, thereby stabilizing the tertiary structure and preventing denaturation.^67^ This effect is enhanced by the abundance of acidic (e.g., glutamic acid and aspartic acid) and basic residues (e.g., lysine and arginine), rather than bulky hydrophobic residues, which facilitates the formation of a tightly bound hydration shell. This shell preserves the conformational mobility required for catalysis under extreme osmotic conditions.^68^ Additionally, such residues may contribute to the stabilization of salt bridges and internal hydrophobic interactions, increasing resistance to salt-induced denaturation.^27,65^

Biotechnological applications of halotolerant xylanases have already been explored across various sectors. For instance, in the processing of marine algae and crustaceans, these enzymes assist in the degradation of xylan-rich polysaccharides, releasing fermentable sugars.^69^ In aquaculture, they may be incorporated into feed formulations for shrimp and fish cultivated in marine environments, improving the digestibility of plant-derived fibers in the diet.^70–72^ In industrial effluent treatment, salt tolerance enables direct enzymatic action in wastewater with high electrical conductivity, eliminating the need for prior desalination.

In summary, the xylanase investigated in this study demonstrated a remarkable capacity to retain activity under extreme NaCl concentrations, positioning it as a promising candidate for industrial applications in hypersaline conditions. The variations observed among the different NaCl concentrations are inconsistent, and there is no clearly defined optimum point. However, the enzyme maintains significant activity even at 6 M NaCl, which emphasizes its halotolerant nature. These findings not only reinforce the enzyme’s biotechnological potential but also open avenues for process optimization in sectors such as food, animal nutrition, bioremediation, and wastewater treatment, encouraging future research into protein engineering strategies aimed at enhancing halotolerance.

### 3.3 Application of the Enzymatic Extract in Sugarcane Bagasse Saccharification

Saccharification is a biotechnological process that converts complex polymers into simpler sugars, enabling their utilization across various industrial sectors, including second-generation ethanol production, animal feed formulation, and agro-industrial waste processing.^73^ In this study, the efficiency of an enzymatic extract was evaluated for the saccharification of sugarcane bagasse, a lignocellulosic residue with high potential for the production of fermentable sugars by testing its application with and without the addition of Ca^2+^ ions at two concentrations (2 and 10 mmol).

The results indicated that the total release of reducing sugars was stable and efficient under all tested conditions (Figure 5). For sucrose, yields of 4.10 ± 0.25 mg/g were obtained with the pure enzymatic extract, and 5.22 ± 0.31 mg/g and 5.66 ± 0.28 mg/g with the addition of 2 and 10 mmol Ca^2+^, respectively, with no statistically significant differences (*p* > 0.05). For glucose, xylose, and other detected sugars, no relevant variations were observed, reinforcing that Ca^2+^ did not exert a marked effect on saccharification efficiency under the conditions employed.

**Figure 5:**
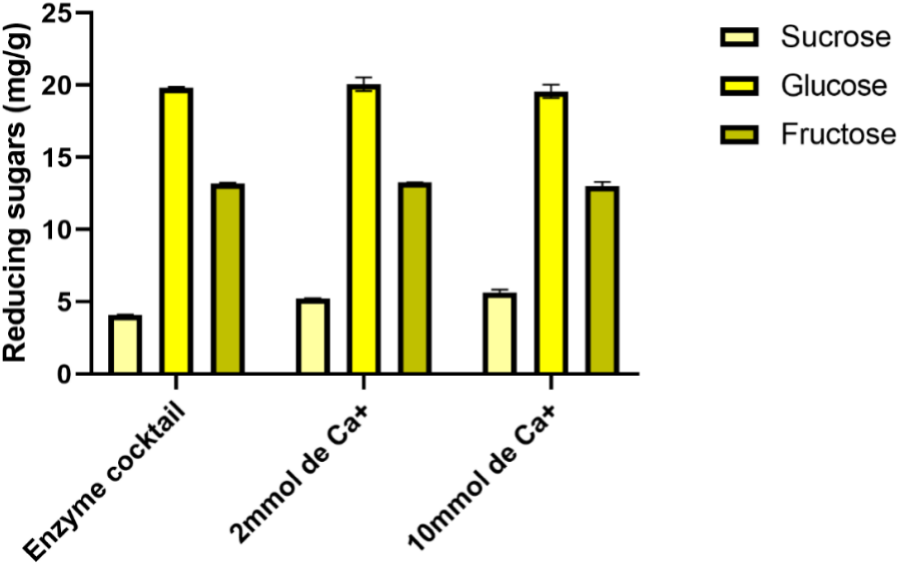
Quantification of reducing sugars (sucrose, glucose, and fructose) released during the enzymatic saccharification of sugarcane bagasse, using the crude (nonpurified) enzymatic extract obtained from *P. roqueforti*. Results are shown for the enzymatic cocktail without salt addition (“Enzyme cocktail”) and after supplementation with 2 and 10 mmol of Ca^+^.

This result contrasts with that reported by,^74^ in which Mg^2+^ acted as a stabilizing cofactor for xylanase, significantly enhancing biomass conversion. The absence of such an effect in the present study may be attributed to the enzyme’s high intrinsic stability, with its active conformation not strongly dependent on metal ion coordination for catalytic activity maintenance.^73^ It is important to highlight that, although the same strain of *P. roqueforti* ATCC 10110 was used in both this study and reference 74, the cultivation conditions and solid substrates differed significantly—specifically, green coconut husk versus yellow mombin residue. These differences are known to influence not only the overall enzyme production levels but also the profile of secreted xylanase isoforms, the degree of glycosylation, and the composition of the enzymatic cocktail found in the crude extract. Consequently, despite both preparations being broadly classified as “xylanase”, it is likely that they have distinct molecular compositions, which may result in varying sensitivities to metal ions such as Mg^2+^ or Ca^2+^. Additionally, since crude extracts were employed, the observed effects of the ions also represent interactions with other enzymes and proteins secreted simultaneously, as well as with soluble components derived from the different substrates. This complexity helps explain the discrepancies noted between the studies.

The favorable performance observed can be explained by the synergistic action of cellulases, hemicellulases, and β-glucosidases, a mechanism previously described by ref.^74^. In this system, cellulases hydrolyze crystalline cellulose fibers, exposing oligosaccharides; hemicellulases cleave hemicellulose branches, releasing sugars such as xylose and arabinose; and β-glucosidases convert cellobiose and terminal oligomers into free glucose, preventing inhibition due to intermediate accumulation. This interaction reduces the need for external additives, improves overall yield, and enhances the economic and environmental viability of the technology.

The yield obtained in this study aligns with the best results reported for commercial enzyme cocktails operating without the addition of metal cofactors,^9,27,74^ thereby expanding its potential for direct application in industrial processes such as cellulosic ethanol production,^74^ formulation of animal feed enriched with simple sugars, and pretreatment of agro-industrial residues for accelerated composting.^56,67^

In summary, the lack of Ca^2+^ dependency combined with the high efficiency of reducing sugar release indicates that the enzymatic cocktail tested is a robust candidate for large-scale biomass conversion processes. Future studies should explore adjustments in enzyme concentration, reaction time, and physicochemical conditions, as well as evaluate its performance on mixed substrates and in continuous systems, thereby bringing the technology closer to industrial deployment.

Thus, considering the observed performance and the potential for optimization under different process conditions, the results obtained provide a solid foundation for advancing the practical application of this enzymatic system. In this context, the xylanase described in this study exhibits promising characteristics for industrial use, particularly because it is produced from an abundant and low-cost agro-industrial residue, contributing to a sustainable approach aligned with the principles of the circular economy. Additionally, production via SSF supports the feasibility of scaling up due to its operational simplicity and lower resource requirements.

The enzyme’s stability under different environmental conditions and its compatibility with biotechnological processes indicate potential for application across various industrial sectors, such as biofuel production, wastewater treatment, and the valorization of lignocellulosic biomass. In this way, the study highlights the enzyme’s practical relevance and its importance for subsequent stages of technological development.

## Supplementary Material



## Notes

Statement of the problem addressed and originality of the approach; contribution of the work to create new knowledge in the field; relevance of the work to advance research and impact to the field of food science and technology (PDF)

## Abbreviations

SSF: Solid-State Fermentation
GCH: Sugarcane Bagasse
PDA: Potato Dextrose Agar
EE: Enzymatic Extract
EDTA: Ethylenediaminetetraacetic Acid
Trolox X-100: *t*-Octylphenoxypolyethoxyethanol
