# Supplementary material for: Solid-State Fermentation of Green Coconut Husk by Penicillium roqueforti for the Production of a Halotolerant Xylanase
Source: ACS Omega. 2026 May 8;11(19):27862–74. doi: 10.1021/acsomega.5c10884 (PMC13191700; doi:10.1021/acsomega.5c10884)
Supplement: Supplementary file 1 [file ao5c10884_si_001.pdf]

1. Statement of the problem addressed and originality of the approach

   This work tackles the pressing environmental challenge of managing and valuing agroindustry waste, with a focus on the bioconversion of coco verde waste (bagaço de coco green, GCH) into high-value xilanase in line with circular economy principles:

- Utilizes Fermentation in Solid State (FES) with *Penicillium roqueforti* ATCC 10110 microorganism to produce xilanase from GCH, a known abundant and underutilized substrate.
- Uses a multivariate statistical optimization strategy (Box-Behnken and Doehlert delineations) to maximize production and characterize the biocatalyst.

2. Contribution of the work to create new knowledge in the field

   The study generates new knowledge on the biochemical properties of xilanases obtained by FES. The main contribution is the discovery of a xilanase extract that stands out for its stability in halotolerância and co-solvents:

- The enzyme exhibits halotolerância, around 70% of residual activity even at an concentration of 6 M of sodium chloride (NaCl).
- The optimized xilanase is stable and active under conditions pH 6.5, 20 °C, and 70 minutes, and exhibits tolerance to ethanol (113.53%), which is crucial for sacarification and fermentation processes (SSF) in the production of biofuels.
- These findings providing valuable information for industrial and food biotechnology.

3. Relevance of the work to advance research and impact to the field of food science and technology

   The work has technological relevance in promoting sustainable bioprocessing strategies and validating the effectiveness of the biocatalyst in a application. The advancement of research in bioprocessing:

- The valorizations of food waste.

- The aplication of the enzyme extract in the surgane bagasse to a release of sacarose, glicose, and frutose, confirming its biotechnological potential for the production of second-generation biocombustibles and animal feed.
- Among the possible uses are food processing:
  Such as juice clarification.
  Aquaculture and animal nutrition, improving the digestibility of fibers in diets.
  Bioremediation and treatment of industrial wastewater with high salinity.
